# Budget impact of endovascular treatment for acute ischaemic stroke patients in the Netherlands for 2015–2021

**DOI:** 10.1007/s12471-023-01788-x

**Published:** 2023-05-12

**Authors:** Lucie A. van den Berg, Olvert A. Berkhemer, Puck S. S. Fransen, Debbie Beumer, Charles B. L. Majoie, Diederik W. J. Dippel, Aad van der Lugt, Robert J. van Oostenbrugge, Wim H. van Zwam, Yvo B. Roos, Marcel G. W. Dijkgraaf, the MR CLEAN investigators

**Affiliations:** 1 Department of Neurology, Amsterdam University Medical Centres, location Academic Medical Centre, Amsterdam, The Netherlands; 2 Department of Epidemiology and Data Science, Amsterdam University Medical Centres, location Academic Medical Centre, Amsterdam, The Netherlands; 3 Amsterdam Neuroscience Research Institute, Neurovascular disorders research programme, Amsterdam University Medical Centres, location Academic Medical Centre, Amsterdam, The Netherlands; 4 Amsterdam Cardiovascular Sciences Research Institute, Atherosclerosis and Ischemic syndromes, Amsterdam University Medical Centres, location Academic Medical Centre, Amsterdam, The Netherlands; 5 Microcirculation research programme, Amsterdam University Medical Centres, location Academic Medical Centre, Amsterdam, The Netherlands; 6 Amsterdam Public Health Research Institute, Methodology research programme, Amsterdam University Medical Centres, location Academic Medical Centre, Amsterdam, The Netherlands; 7 Department of Radiology and Nuclear Medicine, Amsterdam University Medical Centres, location Academic Medical Centre, Amsterdam, The Netherlands; 8 Departments of Neurology, Erasmus University Medical Centre Rotterdam, Rotterdam, The Netherlands; 9 Radiology and Nuclear Medicine, Erasmus University Medical Centre Rotterdam, Rotterdam, The Netherlands; 10 Departments of Neurology, Maastricht University Medical Centre, Maastricht, The Netherlands; 11 Radiology and Nuclear Medicine, Maastricht University Medical Centre, Maastricht, The Netherlands; 12 Cardiovascular Research Institute Maastricht, Maastricht, The Netherlands

**Keywords:** Ischaemic Stroke, Endovascular Procedures, Thrombectomy, Economic Evaluation

## Abstract

**Introduction:**

Endovascular treatment (EVT) has been proven to be both effective and cost-effective for patients with acute ischaemic stroke. We investigated the budget impact of large-scale implementation of EVT for acute ischaemic stroke patients in the Netherlands for 2015–2021.

**Methods:**

An analysis was performed from a healthcare perspective as a preplanned substudy of the Multicenter Randomized Clinical trial of Endovascular Treatment for Acute Ischemic Stroke in the Netherlands (MR CLEAN). Estimated yearly costs during follow-up after stroke for patients who had or had not been treated with EVT as add-on to usual care were linked to numbers of new patients retrieved from 2 Dutch registries of EVT that started after the last inclusion in MR CLEAN (2014). Aggregated costs and costs per care sector were calculated based on prevalence using a population dynamic tool.

**Results:**

From 2015, the yearly number of new acute ischaemic stroke patients receiving EVT increased almost threefold, from 812 in 2015 to 2,370 in 2021. The introduction of EVT plus usual care resulted in estimated net annual savings that increased from € 2.9 million in 2015 to € 58 million in 2021.

**Conclusion:**

Offering EVT as add-on to usual care for acute ischaemic stroke patients was increasingly cost saving from a national healthcare perspective but affected distinct healthcare sectors differently.

**Supplementary Information:**

The online version of this article (10.1007/s12471-023-01788-x) contains supplementary material, which is available to authorized users.

## What’s new?

- Since 2015, endovascular treatment (EVT) is standard care in the Netherlands for patients with acute ischaemic stroke (AIS) caused by large-vessel occlusion.
- The yearly number of AIS patients receiving EVT increased from 812 in 2015 to 2,370 in 2021.
- The estimated mean per patient costs for EVT ranged from € 70.107 in the first year to € 16.514 in years 3–6 and for usual care alone from € 73.760 to € 20.862.
- The introduction of EVT as add-on to usual care resulted in net health care budget savings of € 2.9 million in 2015 to € 58 million in 2021.

## Introduction

To date, 2 acute treatment strategies for acute ischaemic stroke (AIS) patients are available: intravenous thrombolysis (IVT) and endovascular treatment (EVT) [1, 2]. Both therapies are aimed at improving clinical outcome by early opening of the occluded vessel to restore blood flow to the salvageable ischaemic brain tissue that is not already infarcted. There are well-recognised contraindications for IVT, and this treatment is less effective in opening large-vessel occlusions compared with EVT [3, 4].

In 2015, the Multicenter Randomized CLinical trial of Endovascular treatment for Acute ischemic stroke in the Netherlands (MR CLEAN) was the first trial to show clinical effectiveness of EVT, followed by 5 other positive trials, which further supported clinical evidence [5]. The clinical benefit of EVT has been proven to be substantial, with a number needed to treat < 3 for improved functional outcome [2]. As such, EVT has been adopted in international guidelines as standard acute stroke care [6]. Subsequently, the next challenge was to implement EVT for AIS safely on a large scale. To do so, evidence of cost-effectiveness is crucial to guide reimbursement decisions. Recently, we performed an economic evaluation from a societal perspective with a 2-year time horizon alongside the MR CLEAN trial, which showed that EVT improved health and saved costs, thus dominating standard treatment [7].

To further inform healthcare policy makers, we aimed to investigate the impact of large-scale implementation on the healthcare budget. In the current study, we conducted a budget impact analysis (BIA) of EVT in the Netherlands from a healthcare perspective for the years 2015–2021. The results may guide reimbursement decisions and may influence price and volume negotiations between insurance companies and healthcare providers.

## Methods

### Setting and time horizon

The BIA was performed as part of the economic evaluation of MR CLEAN and its extended follow-up study. The study design, methods and results of MR CLEAN, the long-term extension study and the cost-effectiveness and cost-utility analysis have been published elsewhere ([8, 9]; see Table S2 in Electronic Supplementary Material). The current analysis addressed the impact of introducing EVT for AIS patients in the Netherlands as add-on treatment to usual care on the healthcare budget. The budget impact was assessed for the first 7 calendar years (2015–2021) following completion of the MR CLEAN patient inclusion and publication of the positive clinical results, which stimulated nationwide dissemination and implementation [5].

### Perspective, comparison and numbers of patients

**Fig. 1:**
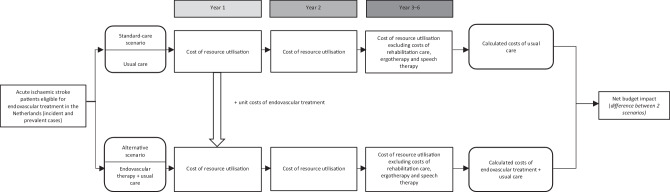
Overall budget impact structure. Budget impact model estimated nationwide impact of endovascular treatment as add-on to usual care on costs of healthcare for eligible acute ischaemic stroke patients (2015–2021). ^ Unknown mean costs for 7th follow-up year of new patients in 2015 have not been included in calendar year 2021

The BIA was performed from a Dutch healthcare perspective. We distinguished 2 major healthcare settings: *institutional care* by hospitals, rehabilitation centres or nursing homes and *noninstitutional care* by general practitioners (GPs), paramedics or home-care organisations. Budget impact was assessed by comparing the estimated yearly costs of institutional and noninstitutional care for Dutch patients who received EVT on top of usual care in the 2015–2021 period versus healthcare costs they would have generated if patients received usual care alone (Fig. 1).

The MR CLEAN Registry study was set up to register all patients in the Netherlands who underwent EVT after the last MR CLEAN inclusion in 2014 [10]. Inclusion in the registry was terminated in December 2018. From 2019 until 2021, we retrieved data from the Dutch Acute Stroke Audit. This is a clinical audit regarding stroke care for patients with AIS in the Netherlands in which all consecutive stroke patients are registered, including data on EVT [11]. From both registries, yearly patient counts for the 2015–2021 period were available. In 2015, EVT was provided to 812 patients; the number of patients increased to 1,138 (2016), 1,478 (2017), 1,712 (2018), 2,233 (2019), 2,322 (2020) and 2,370 (2021).

Budget impact was assessed per calendar year and the analysis was prevalence-based without a half-year correction, meaning that estimated healthcare costs during the first and subsequent years of follow-up for distinct patients were assigned to the calendar year in which each patient’s follow-up year started.

### Cost components, costs and costs per follow-up year of patients

The costs of institutional care included the costs of care in the hospital, rehabilitation centre and/or nursing home. Hospital costs included costs of acute interventions (EVT and IVT), other interventional and diagnostic procedures, inpatient stay (regular admission days and intensive care unit days) and consultations with the medical specialist. EVT as evaluated in the MR CLEAN trial was a new treatment modality in the Netherlands, and its unit costs were determined by detailed precalculation of mean use of personnel, materials and overhead (nearly € 10,000) (reported as part of our economic evaluation) [7]. The costs of rehabilitation included inpatient stay as well as day care. Nursing home costs reflected the costs of inpatient stay.

The costs of noninstitutional care included the costs of GP care, paramedical care and formal home care. GP care costs reflected the costs of GP visits. Paramedical care costs included the costs of consultations provided by physiotherapists, occupational therapists and speech therapists. Formal home-care costs included hourly costs of regular help, personal care and nursing care.

A full account of the measurements and valuation of healthcare resource utilisation, respectively costs can be found in the methods section of the MR CLEAN cost-effectiveness and cost-utility analysis [7]. From that economic evaluation, the estimated mean healthcare costs during the first 2 years of patient follow-up were derived. Undiscounted costs during follow-up in euros were used for the BIA with calendar year 2018, midway the 2015–2021 period, as the base year for costing after general consumer price indexing (cumulative multiplier for 2014–2018: 1.04054) [12]. Incident patients during 2015–2021 were assumed to have generated the mean healthcare costs during the first and second years of follow-up of the patients in the intervention group of the MR CLEAN extension study (Fig. 1). If they would not have received EVT, we assumed that they would have generated the mean healthcare costs during the first and second years of follow-up for patients in the control group (Fig. 1).

For nonobserved mean healthcare costs per patient in follow-up years 3–6 (Fig. 1), we assumed that a patient’s health status would gradually stabilise during the first or the second year following stroke, and that rehabilitation efforts and paramedical care by occupational therapists or speech therapists would be terminated before the end of the second year because of goal achievement or expected lack of further improvement (after consultation of experts in the field). Contrarily, physiotherapy might still be continued as a maintenance therapy for muscle strength and mobility. It was therefore assumed that healthcare costs minus costs of rehabilitation, occupational therapy and speech therapy in the second year of follow-up would reasonably reflect the yearly costs in subsequent follow-up years 3–6.

All mean costs per follow-up year derived from the economic evaluation were calculated by dividing the total healthcare costs per follow-up year by the original number of patients in the study arms, thus including deceased patients [7]. Most patients in the study died within the first year of follow-up, which was followed by a gradually diminishing mortality risk [13]. Furthermore, the extrapolation of mean costs during the second year of follow-up to subsequent years is limited to the sixth follow-up year. We therefore choose to ignore explicit modelling of mortality rates over time.

### Assessment tool

A simple population dynamic model was developed in Microsoft Excel, linking the derived mean healthcare costs by observed numbers of new patients during their years of individual follow-up under the standard-care scenario or under the alternative EVT scenario to the calendar years of the budget impact period. We report the budget impact at the aggregated levels of institutional and noninstitutional care based on yearly patient numbers and mean cost estimates. The budget model is available upon request to allow assessments of budget impact at more local levels relevant to a specific market share or to explore the influence of uncertainty in the cost estimates. To the latter end, 95% confidence intervals for mean costs were generated after bias correction by accelerated nonparametric bootstrapping, drawing 1,000 samples of the same sizes as the original samples of the MR CLEAN study groups, with replacement.

## Results

### Costs during years of follow-up

The estimated mean yearly costs of institutional care for patients who received EVT in the MR CLEAN intervention group were € 60,146 in the first and € 12,856 in the second year of follow-up (Tab. 1). For the third to sixth year of follow-up, the mean yearly costs were assumed to level at € 8,226 (costs of the second year minus rehabilitation costs). The estimated mean yearly costs of noninstitutional care in the intervention group equalled € 9,962 in the first year, € 8,740 year in the second and € 8,288 in subsequent years.

**Table 1:** Mean per patient costs of healthcare by treatment scenario, sector and follow-up year after acute ischaemic stroke^a^

Alternative scenario: EVT plus usual care			
**Care sector**	Follow-up year at patient level		
	**1**	**2**	**3–6**
**Institutional care**	60,146 (55,317–64,863)	12,856 (10,487–15,473)	8,226 (6,268–10,640)
Hospital	19,922 (18,983–20,780)	1,297 (10,17–1,572)	1,297 (1,017–1,572)
Rehabilitation centre	29,363 (25,985–32,635)	4,630 (3,466–5,873)	–
Nursing home	10,860 (8,890–13,177)	6,929 (5,042–9,238)	6,929 (5,042–9,238)
**Non–institutional care**	9,962 (82,230–11,871)	8,740 (7,238–10,345)	8,288 (6,873–9,803)
GP	185 (154–221)	152 (126–180)	152 (126–180)
*Paramedics*	1,599 (1,338–1,886)	1,219 (1,027–1,452)	767 (629–928)
– Physiotherapy	908 (757–1,083)	767 (629–928)	767 (629–928)
– Occupational therapy	368 (284–458)	176 (135–223)	–
– Speech therapy	322 (249–409)	276 (206–364)	–
Home care	8,178 (6,578–9,910)	7,370 (6,099–8,835)	7,370 (6,099–8,835)
**Total**	**70,107 (64,436–76,076)**	**21,597 (18,428–25,206)**	**16,514 (13,896–19,625)**
Standard care scenario: usual care alone			
**Care sector**	Follow-up year at patient level		
	**1**	**2**	**3–6**
**Institutional care**	62,214 (57,010–67,209)	19,276 (15,825–22,999)	10,040 (8,223–12,170)
Hospital	11,004 (10,110–11,979)	1,873 (1,450–2,354)	1,873 (1,450–2,354)
Rehabilitation centre	37,520 (33,849–41,432)	9,236 (6,885–12,055)	–
Nursing home	13,690 (11,827–15,635)	8,167 (6,391–10,205)	8,167 (6,391–10,205)
**Non–institutional care**	11,546 (9,891–13,186)	11,520 (9,726–13,211)	10,822 (9,146–12,411)
GP	138 (114–162)	147 (124–173)	147 (124–173)
*Paramedics*	20,62 (1,748–2,388)	1,798 (1,514–2,076)	1,100 (938–1,251)
– Physiotherapy	1,193 (1,026–1,382)	1,100 (938–1,251)	1,100 (938–1,251)
– Occupational therapy	415 (314–534)	280 (207–376)	–
– Speech therapy	454 (356–568)	418 (320–520)	–
Home care	93,46 (7,811–10,881)	9,575 (7,949–11,140)	95,75 (7,949–11,140)
Total	**73,760 (67,796–79,391)**	**30,796 (26,573–35,398)**	**20,862 (18,140–23,688)**

Data are mean (lower–upper 95% confidence limits)

*EVT* endovascular treatment, *GP* general practitioner

^a^Costs (in euros) are undiscounted. Costing base year was 2018

The mean yearly costs of institutional care for patients receiving usual care in the MR CLEAN control group were € 62,214 in the first, € 19,276 in the second year of follow-up, and an assumed yearly € 10,040 in subsequent years (Tab. 1). For noninstitutional care, the mean yearly costs were € 11,546 in the first year and € 11,510 in the second year and assumed to level at € 10,822 in subsequent years. Tab. 1 reports further details by treatment scenario, care sector and follow-up year.

### Budget impacts

The almost tripling increase—from 812 in 2015 to over 2,300 in 2020–2021—of new yearly patients who received add-on EVT instead of usual care (including IVT) increased pressure on hospital care budgets up to € 16 million yearly or almost € 92 million during 2015–2021. GPs experienced a more limited pressure with an increase of demand for care up to an extra € 156,000 in 2021. Budget savings were noted in all other relevant care sectors. In descending order, the budgets for rehabilitation were mostly affected (−€ 143 million in total), followed by formal home care (−€ 75 million), nursing home care (−€ 68 million) and paramedical care (−€ 17 million).

**Fig. 2:**
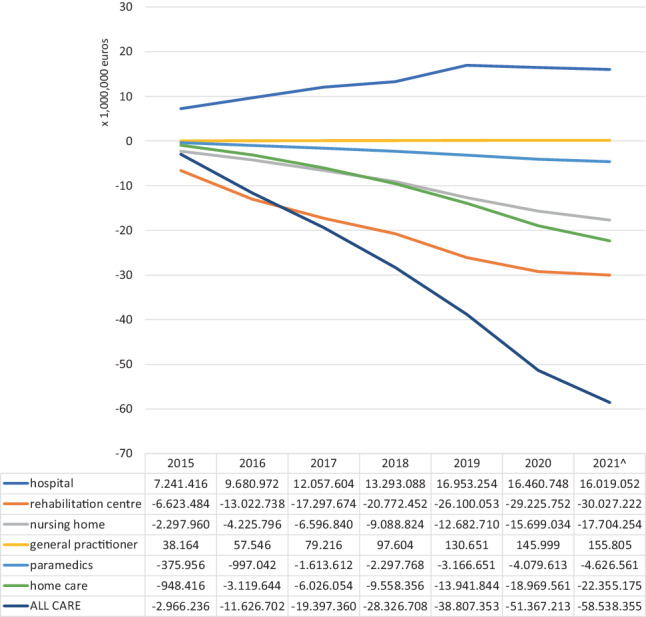
Budget impact of endovascular treatment as add-on to usual care (2015–2021)

Figure 2 shows the budget impact per care sector for the successive calendar years 2015–2021. The yearly net reduction in budget across all care sectors first exceeded the € 50 million mark in 2020 and further continued to increase (Fig. 2).

## Discussion

We estimated the budget impact of EVT for AIS patients in the Netherlands from 2015 through 2021, following the initially and later confirmed positive results of the MR CLEAN study. In 2022, we published the results of our economic evaluation, including a cost-effectiveness analysis [7]. It showed that EVT dominated standard treatment with $ 18,233 saved per extra patient with a good outcome and $ 105,869 saved per additional quality-adjusted life year. In other words, EVT is cost-effective: it improves quality of life and saves costs compared with usual care [7].

In this additional budget impact analysis, we showed that the introduction of EVT as an add-on to usual care already started to pay off by net budget savings of well over € 50 million yearly. These results leave room for facing financial challenges or for additional expenditures in other areas of medicine where these are needed most. Furthermore, it may guide reimbursement decisions and influence future price and volume negotiations between for example insurance companies and healthcare providers.

We did not identify any other BIA of EVT for AIS patients in the literature to compare our results with. One previous Dutch study estimated future healthcare costs by considering the expected increase of stroke patients and a nationwide implementation of acute stroke services [14]. The investigators estimated that the total costs of stroke based on current practice at that time increased from 1.62 billion euros in 2000 to 2.08 billion euros in 2020, taking into account the effect of demographic changes and trends in major risk factors for stroke. Implementing stroke services in 2020 would result in reduction of stroke costs by 13%, to a total of 1.81 billion euros [14]. Although these results are not directly comparable to our study, they also showed stroke is a disease with a heavy burden on total healthcare costs and that implementation of effective treatment strategies significantly results in cost reduction in the healthcare budget. Our findings further expand on these results, as EVT will be part of optimisation and implementation of acute stroke services.

### Strengths and limitations

Our BIA has several strengths and limitations. The current BIA input data were retrieved from our economic evaluation that gathered empirical data on the use of resources following either EVT or usual care during 2 years of follow-up, alongside a pragmatic randomised clinical trial. Hence, results were based on real-life scenarios derived from a single source instead of data based on extrapolation of assumptions from multiple sources, which are often used in economic studies.

We did, however, make assumptions on the patient’s health status and related costs after the 2 years of follow-up to estimate the costs in subsequent years, without performing a sensitivity analysis for different scenarios. The latter may have resulted in considerable changes in the estimated outcomes. Still, given the large amount of net savings, together with a low rate of recurrent strokes or major complications observed during clinical follow-up, it is to be expected such additional analysis would not have changed the results substantially [13]. In addition, our model was based on observed rather than expected stroke patients, who proved eligible for EVT by having received treatment and being included in nationwide Dutch registries. Hence, epidemiological uncertainty was absent.

The current BIA did not address the implementation costs of EVT to attain a geographically optimal spread across the nation beyond the co-operation of study centres already in place. Currently, the optimalisation and organisation of centralisation of EVT is ongoing. As the positive results of the trials were only relatively recently known and Dutch hospitals are still in the process of improving their logistics for EVT, it seems certain that patient numbers may continue to rise in the years to come.

## Conclusion

In view of the yearly numbers of AIS patients who received EVT in the Netherlands from 2015 through 2021, introduction of EVT as add-on to usual care will continue to lead to substantial net annual budget savings.

## Supplementary Information

**Table S1** List of additional MR CLEAN collaborators and their affiliations

**Table S2** Study design, methods and results of MR CLEAN trial its long-term extension study
