# Supplementary material for: Budget impact of endovascular treatment for acute ischaemic stroke patients in the Netherlands for 2015–2021
Source: Neth Heart J. 2023 May 12;31(6):254–9. doi: 10.1007/s12471-023-01788-x (PMC10188812; doi:10.1007/s12471-023-01788-x)
Supplement: Supplementary file 1 — Table S1 List of additional MR CLEAN collaborators and their affiliations [file 12471_2023_1788_MOESM1_ESM.docx]

**Table S1** List of additional MR CLEAN collaborators and their affiliations

Albert J. Yoo, M.D.,8 Wouter J. Schonewille, M.D.,9 Jan Albert Vos, M.D., Ph.D.,10 Paul J. Nederkoorn, M.D., Ph.D.,5 Marieke J.H. Wermer, M.D., Ph.D.,11 Marianne A.A. van Walderveen, M.D., Ph.D.,12 Julie Staals, M.D., Ph.D.,4 Jeannette Hofmeijer, M.D., Ph.D.,13 Jacques A. van Oostayen, M.D., Ph.D.,14 Geert J. Lycklama à Nijeholt, M.D., Ph.D.,15 Jelis Boiten, M.D., Ph.D.,16 Patrick A. Brouwer, M.D.,3 Bart J. Emmer, M.D., Ph.D.,1 Sebastiaan F. de Bruijn, M.D., Ph.D.,17 Lukas C. van Dijk, M.D.,18 L. Jaap Kappelle, M.D., Ph.D.,19 Rob H. Lo, M.D.,20 Ewoud J. van Dijk, M.D., Ph.D.,21 Joost de Vries, M.D., Ph.D.,22 Paul L.M. de Kort, M.D., Ph.D.,23 Jan S.P. van den Berg, M.D., Ph.D.,24 Boudewijn A.A.M. van Hasselt, M.D.,25 Leo A.M. Aerden, M.D., Ph.D.,26 René J. Dallinga, M.D.,27 Marieke C. Visser, M.D., Ph.D.,28 Joseph C.J. Bot, M.D., Ph.D.,29 Patrick C. Vroomen, M.D., Ph.D.,30 Omid Eshghi, M.D., 31 Tobien H.C.M.L. Schreuder, M.D.,32 Roel J.J. Heijboer, M.D.,33 Koos Keizer, M.D., Ph.D.,34 Alexander V. Tielbeek, M.D., Ph.D.,35 Heleen M. den Hertog, M.D., Ph.D.,36 Dick G. Gerrits, M.D., 37 Renske M. van den Berg-Vos, M.D., Ph.D.,38 Giorgos B. Karas, M.D.,39 Ewout W. Steyerberg, M.D., Ph.D.,7 H. Zwenneke Flach, M.D.,26 Henk A. Marquering Ph.D.,40,1 Marieke E.S. Sprengers, M.D., Ph.D.,1 Sjoerd F.M. Jenniskens, M.D., Ph.D.,41 Ludo F.M. Beenen, M.D.,1 René van den Berg, M.D., Ph.D.,1 Peter J. Koudstaal, M.D., Ph.D.,2

1 Department of Radiology, Amsterdam University Medical Centers, location University of Amsterdam, the Netherlands; 2 Department of Neurology, Erasmus MC University Medical Center Rotterdam, the Netherlands; 3 Department of Radiology, Erasmus MC University Medical Center Rotterdam, the Netherlands; 4 Department of Neurology, Maastricht University Medical Center and Cardiovascular Research Institute Maastricht (CARIM), the Netherlands; 5 Department of Neurology, Academic Medical Center Amsterdam, the Netherlands; 6 Department of Radiology, Maastricht University Medical Center, the Netherlands; 7 Department of Public Health, Erasmus MC University Medical Center Rotterdam, the Netherlands; 8 Department of Radiology, Texas Stroke Institute, Texas, United States of America; 9 Department of Neurology, Sint Antonius Hospital, Nieuwegein, the Netherlands; 10 Department of Radiology, Sint Antonius Hospital, Nieuwegein, the Netherlands; 11 Department of Neurology, Leiden University Medical Center, the Netherlands; 12 Department of Radiology, Leiden University Medical Center, the Netherlands; 13 Department of Neurology, Rijnstate Hospital, Arnhem, the Netherlands; 14 Department of Radiology, Rijnstate Hospital, Arnhem, the Netherlands; 15 Department of Radiology, MC Haaglanden, the Hague, the Netherlands; 16 Department of Neurology, MC Haaglanden, the Hague, the Netherlands; 17 Department of Neurology, HAGA Hospital, the Hague, the Netherlands; 18 Department of Radiology, HAGA Hospital, the Hague, the Netherlands; 19 Department of Neurology, University Medical Center Utrecht, the Netherlands; 20 Department of Radiology, University Medical Center Utrecht, the Netherlands; 21 Department of Neurology, Radboud University Medical Center, Nijmegen, the Netherlands; 22 Department of Neurosurgery, Radboud University Medical Center, Nijmegen, the Netherlands; 23 Department of Neurology, Sint Elisabeth Hospital, Tilburg, the Netherlands; 24 Department of Neurology, Isala Klinieken, Zwolle, the Netherlands; 25 Department of Radiology, Isala Klinieken, Zwolle, the Netherlands; 26 Department of Neurology, Reinier de Graaf Gasthuis, Delft, the Netherlands; 27 Department of Radiology, Reinier de Graaf Gasthuis, Delft, the Netherlands; 28 Department of Neurology, VU Medical Center, Amsterdam, the Netherlands; 29 Department of Radiology, Amsterdam University Medical Centers, location ‘’Vrije Universiteit’’ of Amsterdam, Amsterdam, the Netherlands; 30 Department of Neurology, University Medical Center Groningen, the Netherlands; 31 Department of Radiology, University Medical Center Groningen, the Netherlands; 32 Department of Neurology, Atrium Medical Center, Heerlen, the Netherlands; 33 Department of Radiology, Atrium Medical Center, Heerlen, the Netherlands; 34 Department of Neurology, Catharina Hospital, Eindhoven, the Netherlands; 35 Department of Radiology, Catharina Hospital, Eindhoven, the Netherlands 36 Department of Neurology, Medical Spectrum Twente, Enschede, the Netherlands; 37 Department of Radiology, Medical Spectrum Twente, Enschede, the Netherlands; 38 Department of Neurology, Sint Lucas Andreas Hospital, Amsterdam, the Netherlands; 39 Department of Radiology, Sint Lucas Andreas Hospital, Amsterdam, the Netherlands; 40 Department of Biomedical Engineering and Physics, Amsterdam University Medical Centers, location University of Amsterdam, the Netherlands; 41 Department of Radiology, Radboud University Medical Center, Nijmegen, the Netherlands.
