# Supplementary material for: Budget impact of endovascular treatment for acute ischaemic stroke patients in the Netherlands for 2015–2021
Source: Neth Heart J. 2023 May 12;31(6):254–9. doi: 10.1007/s12471-023-01788-x (PMC10188812; doi:10.1007/s12471-023-01788-x)
Supplement: Supplementary file 2 — Table S2 Study design, methods and results of MR CLEAN trial its long-term extension study [file 12471_2023_1788_MOESM2_ESM.docx]

**Table S2** Study design, methods and results of MR CLEAN and its long-term extension study

MR CLEAN was a randomized, multicentre trial in which endovascular treatment plus usual care (intervention group) was compared to usual care alone (control group) in patients with acute ischemic stroke caused by a proximal intracranial arterial occlusion of the anterior circulation.(1) Usual care included best medical management according to national and international guidelines, including intravenous thrombolysis (IVT) with recombinant tissue plasminogen activator. Endovascular treatment consisted of intra-arterial catheterization with a microcatheter to the level of occlusion and delivery of a thrombolytic agent, mechanical thrombectomy, or both. Primary outcome of the trial was functional outcome, assessed by the modified Rankin Scale (mRS) score at 90 days. The mRS is a 7-point scale ranging from 0 (no symptoms) to 6 (dead).(2) A score of 2 points or less indicates functional independence. In total, 500 patients from 16 medical centres in the Netherlands were randomly assigned in MR CLEAN between December 2010 and April 2014, of which 233 in the intervention group and 267 patients to the control group. The absolute difference in the rate of functional independence was 13,5 % (32·6% vs. 19·1%; 95% CI interval, 5·9 to 21·2) in favour of the intervention group, without significant differences in mortality or the occurrence of serious adverse events. At two-year follow-up the distribution of outcomes on the modified Rankin scale (mRS) favoured endovascular treatment over usual treatment similar to the outcome at three months (difference 13.2%, (37,1% vs. 23,9%, 95% CI interval, 4 to 22).(3) At two years, the mean health utility score was 0·48 among patients randomly assigned to endovascular treatment as compared with 0.38 among patients randomly assigned to usual treatment (mean difference, 0·10; 95% CI, 0·03 to 0·16; P=0·006). The cumulative 2-year mortality rate was 26·0% in the intervention group and 31·0% in the control group (adjusted hazard ratio, 0·9; 95% CI, 0·6 to 1·2; P=0·46). For the economic evaluation empirical data on the use of resources were gathered parallel to the MR CLEAN trial up to two years of follow-up.(4) Incremental cost-effectiveness ratios as the extra costs per additional patient with functional independence (mRS 0-2) and the extra cost per quality adjusted life year (QALY) gained were calculated. The mean costs per patient in the intervention group were €91,902 vs. €104,135 in the control group (mean difference €12,234, 95% confidence interval [CI]: €27,690 to €3,964 (base year for unit costing 2014)). Endovascular treatment dominated standard treatment with €13,247 saved per extra patient with a good outcome and €76,937 saved per additional quality adjusted life year (QALY).

**References**

1. Berkhemer OA, Fransen PSS, Beumer D, van den Berg LA, Lingsma HF, Yoo AJ, et al. A randomized trial of intraarterial treatment for acute ischemic stroke. New England Journal of Medicine. 2015;372(1).

2. Banks JL, Marotta CA. Outcomes validity and reliability of the modified rankin scale: Implications for stroke clinical trials - A literature review and synthesis. Vol. 38, Stroke. 2007. p. 1091–6.

3. van den Berg LA, Roos YB. Two-year outcome after endovascular treatment for stroke. New England Journal of Medicine. 2017;376(26).

4. van den Berg LA, Berkhemer OA, Fransen PSS, Beumer D, Lingsma H, Majoie CBM, et al. Economic Evaluation of Endovascular Treatment for Acute Ischemic Stroke. Stroke. 2022;29(2):968–75.
